# Quinoa *CqNLP9*: a possible regulator of nitrogen metabolism under low nitrogen stress

**DOI:** 10.3389/fpls.2026.1787306

**Published:** 2026-03-18

**Authors:** Ruling Xu, Ni An, Xiaoting Chen, Jialing Cao, Yaru Liang, Yating Wu, Liyan Yang, Xueyong Zhou

**Affiliations:** School of Life Science, Shanxi Engineering Research Center of Microbial Application Technologies, Shanxi Normal University, Taiyuan, China

**Keywords:** *CqNLP9*, interaction, low-nitrogen (LN), nitrogen metabolism, quinoa

## Abstract

**Introduction:**

Nitrogen metabolism constitutes a fundamental physiological process governing plant growth and development. Nin-like protein (NLP) transcription factors act as central regulators in nitrate signaling, coordinating nitrogen uptake and assimilation. Quinoa (*Chenopodium quinoa*), known for its adaptability to nutrient-poor soils, serves as an excellent model for dissecting nitrogen-acquisition mechanisms. This study aims to explore the role of *CqNLP9* in quinoa under low-nitrogen (LN) stress and its potential regulatory mechanisms.

**Methods:**

The expression level of *CqNLP9* in quinoa was examined by qRT-PCR after 30 days of LN treatment. Gain- and loss-of-function approaches were employed by overexpressing *CqNLP9* in *Arabidopsis* and silencing its homologous gene *NbNLP9* in tobacco. Phenotypic changes and related physiological and biochemical parameters were analyzed under LN conditions. Additionally, yeast two-hybrid and luciferase complementation imaging assays were performed to identify proteins interacting with CqNLP9.

**Results:**

Following 30 days of LN treatment, *CqNLP9* expression increased 24-fold in quinoa compared with plants grown in complete nutrient solution. Under LN conditions, *CqNLP9*-overexpressing *Arabidopsis* lines exhibited greater biomass, chlorophyll content, soluble protein, and total nitrogen compared with wild-type (Col-0). Activities of key nitrogen metabolism enzymes —nitrate reductase, nitrite reductase, glutamine synthetase, glutamate synthase, and glutamate dehydrogenase —were elevated, along with enhanced peroxidase, superoxide dismutase, and catalase activities. Malondialdehyde content decreased, indicating reduced oxidative damage. Conversely, silencing of the homologous gene *NbNLP9* in tobacco resulted in opposite phenotypic changes. Furthermore, yeast twohybrid and luciferase complementation imaging assays revealed a physical interaction between CqNLP9 and CqMOB1B, a core component of the Hippo signaling pathway.

**Discussion:**

These findings suggest that *CqNLP9* might contribute to improved low-nitrogen tolerance through modulation of nitrogen metabolism enzyme activities, enhancement of ROS scavenging capacity, and maintenance of chlorophyll and soluble protein balance, which in turn could facilitate nitrogen uptake and assimilation. The interaction between CqNLP9 and CqMOB1B offers new perspectives on the crosstalk between nutrient signaling and developmental regulatory pathways in plants.

## 1 Introduction

Nitrogen, a key structural element of biomolecules such as proteins, nucleic acids, and chlorophyll, is actively involved in multiple metabolic processes and serves as an essential nutrient for plant growth and development (Kishorekumar et al., 2020). The application of nitrogen fertilizer in soil is closely correlated with the growth, development, and yield of crops (Fang et al., 2018; Li et al., 2024). However, the prevailing emphasis on higher crop yields has resulted in excessive nitrogen application, leading to substantial nutrient waste and severe environmental pollution, particularly in China (Chen and Liao, 2017; Li et al., 2017; Quan et al., 2020). Consequently, enhancing plant nitrogen uptake capability and improving nitrogen use efficiency are important for sustainable agriculture. Previous studies have demonstrated that the *NLP* (Nin-Like Protein) transcription factor is a key regulator in nitrate signal transduction and nitrogen metabolism, which provides a potential strategy to enhance nitrogen use efficiency (Camargo et al., 2007; Mu and Luo, 2019; Liu et al., 2021; Wang et al., 2025b).

The *NLP* transcription factors contain a conserved RWP-RK domain that binds the nitrate-responsive cis-element (NRE). This binding triggers the transcriptional activation of downstream genes and mediates the primary nitrate response in the nucleus (Liu et al., 2017; Kumar et al., 2018; Liu et al., 2018; Wang et al., 2024). Under low nitrogen stress, NLPs enhance nitrogen use efficiency via regulating nitrogen assimilation through transcriptional and post-translational mechanisms and coordinating metabolic pathways (Konishi and Yanagisawa, 2013). In *Arabidopsis* and legumes, it has been confirmed that *NLP* could specifically bind to NRE and activate the transcription of NRE-dependent nitrate-responsive genes (Feng et al., 2011; Krouk, 2017; Liu et al., 2022). Recent studies have demonstrated that *OsZIP4* regulates the expression of nitrate-responsive genes and modulates nitrate reductase (NR) activity, thereby influencing rice growth and development (Wang et al., 2021). In rice (*Oryza sativa*), overexpression of *OsNLP1* and *OsNLP3* significantly enhance both nitrogen use efficiency and grain yield under varying nitrogen conditions (Alfatih et al., 2020; Zhang et al., 2022). *NLP* transcription factors represent crucial genetic targets for enhancing nitrogen use efficiency in crop plants.

*NLP* transcription factors also participate in photosynthesis and other physiological processes through nitrogen metabolic pathways. In *Arabidopsis*, *AtNLP2* significantly enhances biomass accumulation and promotes vegetative growth while regulating root architecture development (Konishi et al., 2021). *AtNLP7* has been identified as a key regulator of chlorophyll homeostasis, where it activates the nitrate-responsive NLP7-HB52/54-VAR2 pathway under combined high-light and low-nitrogen stress conditions, and optimized photosynthetic energy conversion efficiency (Ariga et al., 2022).

Quinoa (*Chenopodium quinoa*) is a strategic crop valued for its exceptional adaptability to adverse environments, including nitrogen-deficient soils (Cárdenas-Castillo et al., 2021). It is of great significance to mine low-nitrogen adaptive mechanism of quinoa. In previous studies, we conducted transcriptome analysis of quinoa under different nitrogen concentration treatments and discovered that *CqNLP9* shows a strong response under low-nitrogen stress (Yang et al., 2024).

Here, we explored the regulatory function of *CqNLP9* in nitrogen metabolism under low-nitrogen conditions by using *Arabidopsis thaliana* as a model system in combination with TRV-mediated Virus-Induced Gene Silencing (VIGS) technology.

## 2 Materials and methods

### 2.1 Plant material and growth conditions

Seeds of a quinoa line ZK7 were provided by the Crop Research Institute, Shanxi Academy of Agricultural Sciences, *Nicotiana benthamiana* and *Arabidopsis thaliana* (Col-0 accession) were maintained in our laboratory. These seedlings were cultivated in a growth chamber at 200 µmol·m^-2^·s^-1^ intensity, and 75% humidity. The growth temperatures were set at 24°C/20°C (day/night) for quinoa, 22°C/20°C for *Arabidopsis*, and 25°C/22°C for *N. benthamiana*, under a 16 h light/8 h dark photoperiod.

### 2.2 Quinoa treatment, RNA extraction and qRT-PCR analysis

When the quinoa seedlings reached 4–6 leaf stage, the uniform ones were moved to pots (20-cm diameter) filled with sand and watered with complete nutrient (CK) and low-nitrogen solution (LN) as our previous study (Yang et al., 2024). The composition of CK and LN nutrient solutions is shown in **Supplementary Table 1**. Each solution was prepared by dissolving the corresponding reagents in 900 mL of distilled water, followed by adjustment to a final volume of 1,000 mL with distilled water. The pH was maintained at 5-6. The culture solutions were refreshed every 2 or 3 days. After 5 days and 30 days of treatment, leaf samples were collected for gene expression analysis.

Total RNA was extracted from quinoa leaves using TransZol Up Plus RNA Kit (Transgen, China). The *TransScript*^®^ One-Step gDNA Removal and cDNA Synthesis Super Mix (Transgen, China) was used to prepare the cDNA. The primers (F: 5’-TGTTCATCTTAGGAGTTCAG-3’, R: 5’-CAGGTTCTACAGCATCATAA-3’) were designed using NCBI (https://www.ncbi.nlm.nih.gov/tools/primer-blast). The qRT-PCR was performed on the QuantStudio-3 PCR system (Life Technologies, Singapore) using PerfectStart Green qPCR SuperMix (Transgen, China). The amplification parameters for qRT-PCR were 40 cycles with annealing at 60 °C. The quinoa Elongation Factor 1 alpha (*EF1α*) was used as the internal standard gene (Zhu et al., 2021). Relative gene expression level was calculated using the 2^−ΔΔCT^ method (Livak and Schmittgen, 2001) with three dependent replicates were adopted, one replicate comprised tissue pooled from three plants.

### 2.3 Vector construction and *Agrobacterium*-mediated genetic transformation

We performed PCR amplification with 2 × TransFast^®^ Taq PCR SuperMix (TransGen, China) according to the manufacturer’s instruction. The coding sequence (CDS) of *CqNLP9* was amplified from quinoa cDNA using gene-specific primers (F: 5’-ATGGATGATGGTTCCTTTAATCCT-3’; R: 5’-TTAGGATAAACCGCTGCTGCCA-3’). The PCR product was double-digested with *Bam*H І and *Sma* І and ligated into the corresponding sites of the pBI121 vector. The resulting recombinant plasmid pBI121-*CqNLP9* was introduced into *Agrobacterium* strain GV3101 and subsequently transformed into *Arabidopsis thaliana* inflorescences via the floral dip method (Clough and Bent, 1998). Transgenic T1 seeds were selected on MS medium supplemented with 50 mg/L kanamycin. Homozygous T3 *CqNLP9-*OE lines (*Arabidopsis* transgenic lines heterologously expressing *CqNLP9*) were obtained through successive selection.

The conserved sequence of *NbNLP9*, an ortholog of *CqNLP9* in *N. benthamiana*, was identified from the VIGS Tool (https://vigs.solgenomics.net/). Using *N. benthamiana* cDNA as template, the *NbNLP9* conserved sequence with the conserved RWP-RK domain was amplified with primers (F: 5’-AGCATCAGTTTGCAGGTACTC-3’; R: 5’-GATTCAGTCCATGGTGCCGAG-3’). The PCR product was digested with *Xba* І and *Bam*H І, then ligated into the corresponding sites of the pTRV2 vector to generate the pTRV2-*NbNLP9* silencing construct. The recombinant plasmid was introduced into *Agrobacterium* strain GV3101 for the generation of *NbNLP9*-silenced tobacco.

The *CqNLP9* was cloned into pSUPER1300-GFP for subcellular localization analysis. Protein interactions were predicted via the STRING database (https://cn.string-db.org/) using the AtNLP2 (the ortholog of CqNLP9) as query sequence, and CqMOB1B was identified. To validate the interaction between CqNLP9 and CqMOB1B, we constructed pGBKT7-*CqNLP9* and pGADT7-*CqMOB1B* vectors. In the yeast two-hybrid (Y2H) assays, the pGBKT7 vector served as the negative control, while the combination of pGADT7-*T* and pGBKT7–*53* was used as the positive control. Additionally, we used pCAMBIA1300-nLuc*-CqNLP9* and pCAMBIA1300-cLuc-*CqMOB1B* for luciferase complementation (LUC) assays.

### 2.4 Low nitrogen treatment for *CqNLP9*-OE *Arabidopsis* and *NbNLP9*-silenced tobacco

Homozygous T3 seedlings from *CqNLP9*-OE lines and wild-type seedlings were cultivated for 4 weeks. Subsequently, they were treated with a complete nutrient solution (CK) and a low-nitrogen solution (LN) for 30 days (**Supplementary Table 1**).

Similarly, *NbNLP9*-silenced tobacco and wild-type tobacco were grown to the 4–6 leaf stage and then exposed to CK and LN solution for 30 days.

### 2.5 Biomass assay

Seedling growth profile under LN and CK treatments were compared. Ten *Arabidopsis* and tobacco seedlings were randomly collected from each treatment group on the 30th day of treatment. The fresh weight and dry weight of seedling roots and shoots were recorded, and plant height and root length were measured. Three independent replicates were adopted, one replicate represented 10 single plant from each group.

### 2.6 Determination of chlorophyll and soluble protein content

Thirty days after LN treatment, chlorophyll content was measured spectrophotometrically according to established methods (Wassie et al., 2020). The soluble protein content was determined using the Coomassie Brilliant Blue G-250 staining method (Ernst and Zor, 2010). Three independent replicates were used, one replicate consisted of pooled leaf tissue from five individual plants grown in the same tray.

### 2.7 Nitrogen content and nitrogen metabolism-related enzyme activity

Thirty days after LN treatment, nitrogen content was quantified with a MACRO Cube Elemental Analyzer (Elmentar, Germany). Nitrate concentration was determined spectrophotometrically by the salicylate method (Cataldo et al., 1975). Enzyme activities were determined using commercial assay kits (Abbkine, China) according to the manufacturer’s protocols Nitrate reductase (NR): Cat. No. KTB4016; Nitrite reductase (NiR): Cat. No. KTB4017; Glutamine synthetase (GS): Cat. No. KTB3042; Glutamate synthase (GOGA): Cat. No. KTB3040; Glutamate dehydrogenase (GDH): Cat. No. KTB3041. There were three independent replicates for each treatment, one replicate consisted of pooled leaf tissue from five individual plants.

### 2.8 Measurement of antioxidant enzyme activity and MDA content

Thirty days after LN treatment, antioxidant enzyme activity and malondialdehyde (MDA) content were analyzed according to the following methods. The guaiacol method was employed to measure the activity of peroxidase (POD). The activity of superoxide dismutase (SOD) was determined using the nitro blue tetrazolium (NBT) method (Sun et al., 2013). The activity of catalase (CAT) was assessed via spectrophotometry (Kang et al., 2003). The thiobarbituric acid (TBA) method was applied to quantify the content of MDA (Tsikas, 2017). Three independent replicates were adopted for each physiological indices, one replicate consisted of pooled leaf tissue from five individual plants.

### 2.9 Subcellular localization of the *CqNLP9*

The pSUPER1300-GFP-*CqNLP9* construct was introduced into *Agrobacterium* GV3101. Equal volumes (OD_600_ = 0.5) of the transformed bacteria liquid and pSUPER2300-*H2B*-mCherry control were co-infiltrated into leaves of 4-week-old *N. benthamiana*. After a 48-hour dark treatment, the samples were observed under a laser scanning confocal microscope (Zeiss, Germany).

### 2.10 Protein interaction between *CqNLP9* and *CqMOB1B*

The bait (pGBKT7-*CqNLP9*) and prey (pGADT7-*CqMOB1B*) vectors were co-transformed into Y2HGold yeast cells via the lithium acetate method. Transformants were selected on SD/-Leu/-Trp plates, and positive colonies were cultured in SD/-Leu/-Trp liquid medium to mid-log phase (OD_600_ = 0.8-1.0). Serial 10-fold dilutions (10^-1^-10^-4^) were spotted onto quadruple dropout plates (SD/-Leu/-Trp/-His/-Ade) containing X-α-Gal. Protein interactions were assessed by blue colony development after 3–5 days at 30 °C.

The pCAMBIA1300-nLuc-*CqNLP9* and pCAMBIA1300-cLuc-*CqMOB1B* constructs were introduced into *Agrobacterium* GV3101. Bacterial suspensions (OD_600_ = 0.5) were mixed 1:1 and infiltrated into *N. benthamiana* leaves. After 48 h dark adaptation, leaves were sprayed with 0.5 mM D-luciferin and dark-adapted for 15 min before imaging with an *in vivo* molecular imaging system.

### 2.11 Data analysis

Data are presented as mean ± standard deviation (SD) from at least three independent experiments. The differences among groups were analyzed using two-way analysis of variance (ANOVA) and Duncan’s multiple range test was applied. For all analyses, statistical significance was set at P < 0.05. Significance levels are denoted as follows: *P < 0.05, **P < 0.01, ***P < 0.001, ****P < 0.0001; ns, not significant. All analyses were conducted using GraphPad Prism software (version 9.0).

## 3 Results

### 3.1 *CqNLP9* expression profile under different nitrogen concentrations and gene cloning

**Figure 1:**
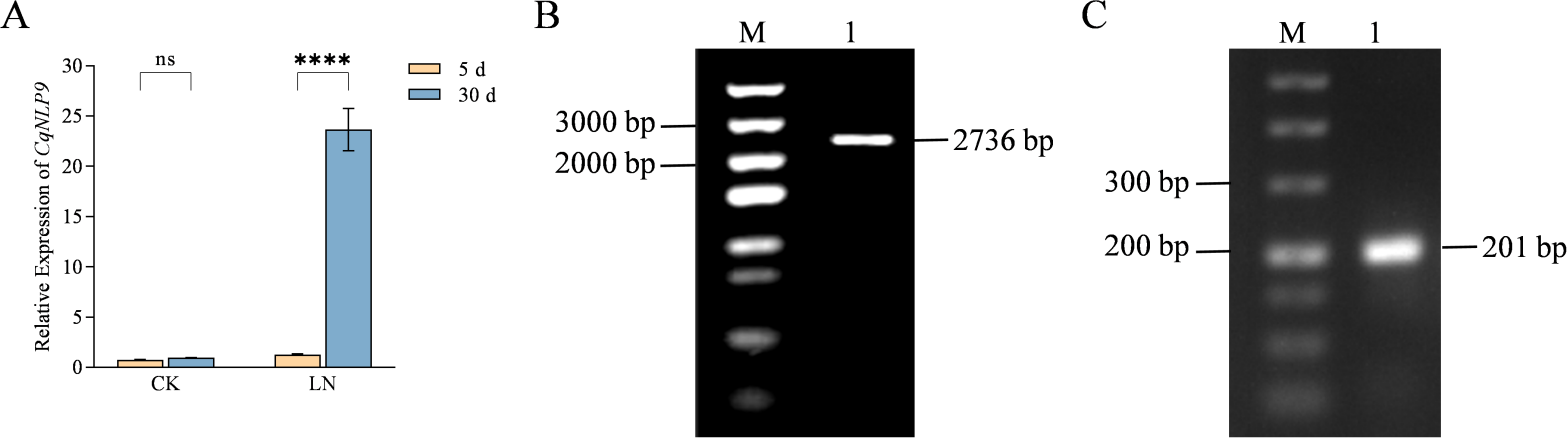
*CqNLP9* expression profile under low-nitrogen stress, cloning of *CqNLP9 and NbNLP9*. **(A)** Expression levels of quinoa *CqNLP9* under different treatments. Quinoa seedlings were grown under complete nutrient solution (CK) or low nitrogen solution (LN) for 5 days and 30 days. Data are presented as means ± SD. Each biological replicate (n = 3 independent replicates) consisted of pooled tissues from three individual plants per treatment per time point. Statistical analysis was performed using two-way ANOVA followed by Duncan’s multiple range test. ****P < 0.0001 indicates a significant difference between 5 d and 30 d within the same treatment group (CK or LN). **(B)** *CqNLP9* cloning. M: 5,000 bp DNA Ladder, and the cloned product is the *CqNLP9* CDS sequence, which is used to construct a plant overexpression vector. **(C)** Cloning of the conserved sequence of *NbNLP9*, an ortholog gene of *CqNLP9*. M: 2,000 bp DNA Ladder, and the cloned product is used to construct a tobacco silencing vector.

*CqNLP9* gene expression was dynamically regulated by nitrogen availability (**Figure 1A**). After 5 days of treatment, no significant differences were detected between the control (CK) and low nitrogen (LN) groups. In contrast, at 30 days, the expression level of *CqNLP9* in the LN group was significantly upregulated, reaching 24-fold that of the CK, suggesting its potential involvement in nitrogen limitation response.

We then cloned coding sequence (CDS) of *CqNLP9* (2,736-bp) and a 201-bp conserved fragment of ortholog *NbNLP9* (**Figures 1B, C**) and generated pBI121-*CqNLP9* and pTRV2-*NbNLP9* constructs (**Supplementary Figures 1A, B**).

### 3.2 Overexpression of *CqNLP9* enhances nitrogen metabolism in *Arabidopsis thaliana*

**Figure 2:**
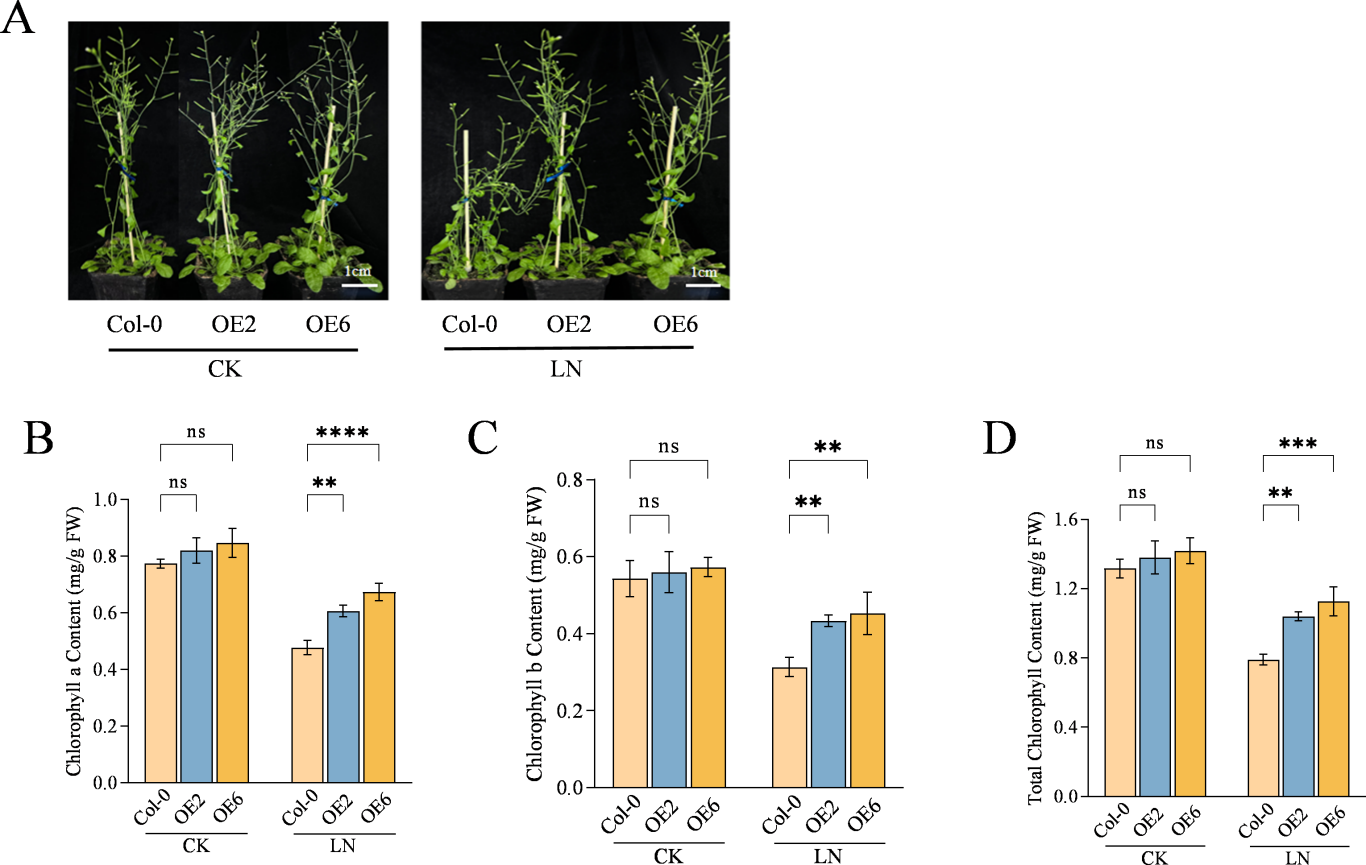
Overexpression of *CqNLP9* promotes the seedling growth and the chlorophyll content in *Arabidopsis thaliana*. **(A)** Growth profile of wild-type *Arabidopsis* (Col-0) and *CqNLP9-*OE lines (OE2 and OE6). Three independent replicates were adopted, one replicate represented 10 single plant from each group. Scale bar = 1 cm. **(B)** Chlorophyll a content. **(C)** Chlorophyll b content. **(D)** Total chlorophyll content. CK, Complete nutrient solution; LN, Low nitrogen solution. Data are presented as means ± SD. Each biological replicate (n = 3 independent replicates) consisted of pooled leaf tissues from five individual plants per line per treatment. Statistical analysis was performed using two-way ANOVA followed by Duncan’s multiple range test. Asterisks indicate significant differences between the Col-0 and overexpression line (OE2 or OE6) within the same treatment group (**P < 0.01, ***P < 0.001, ****P < 0.0001; ns, not significant).

To investigate the function of *CqNLP9*, we heterologously overexpressed *CqNLP9* in *Arabidopsis thaliana* and obtained transgenic lines OE2 and OE6, as verified by RT-PCR (**Supplementary Figures 2A, B**). After 30 days of LN treatment, wild-type (Col-0) plants showed significant growth inhibition, whereas transgenic lines OE2 and OE6 exhibited approximately 30% greater plant height, 1.4-fold longer roots, and significantly increased shoot and root weight compared to those of Col-0 (**Figure 2A**; **Supplementary Table 2**). Heterologous expression of *CqNLP9* confers LN tolerance in *Arabidopsis*, suggesting a conserved function that may extend to quinoa. To investigate the function of *CqNLP9* in photosynthesis under low-nitrogen (LN) stress, chlorophyll content was measured. Under control (CK) conditions, chlorophyll a, chlorophyll b, and total chlorophyll levels did not differ significantly between wild-type (Col-0) and transgenic lines OE2 and OE6. After 30 days of LN treatment, all genotypes showed reduced chlorophyll content relative to CK. Col-0 exhibited the largest decrease, while OE2 and OE6 maintained significantly higher chlorophyll levels under LN (**Figures 2B–D**). These results indicate that *CqNLP9* helps mitigate chlorophyll degradation under nitrogen deficiency and helps preserve photosynthetic function.

**Figure 3:**
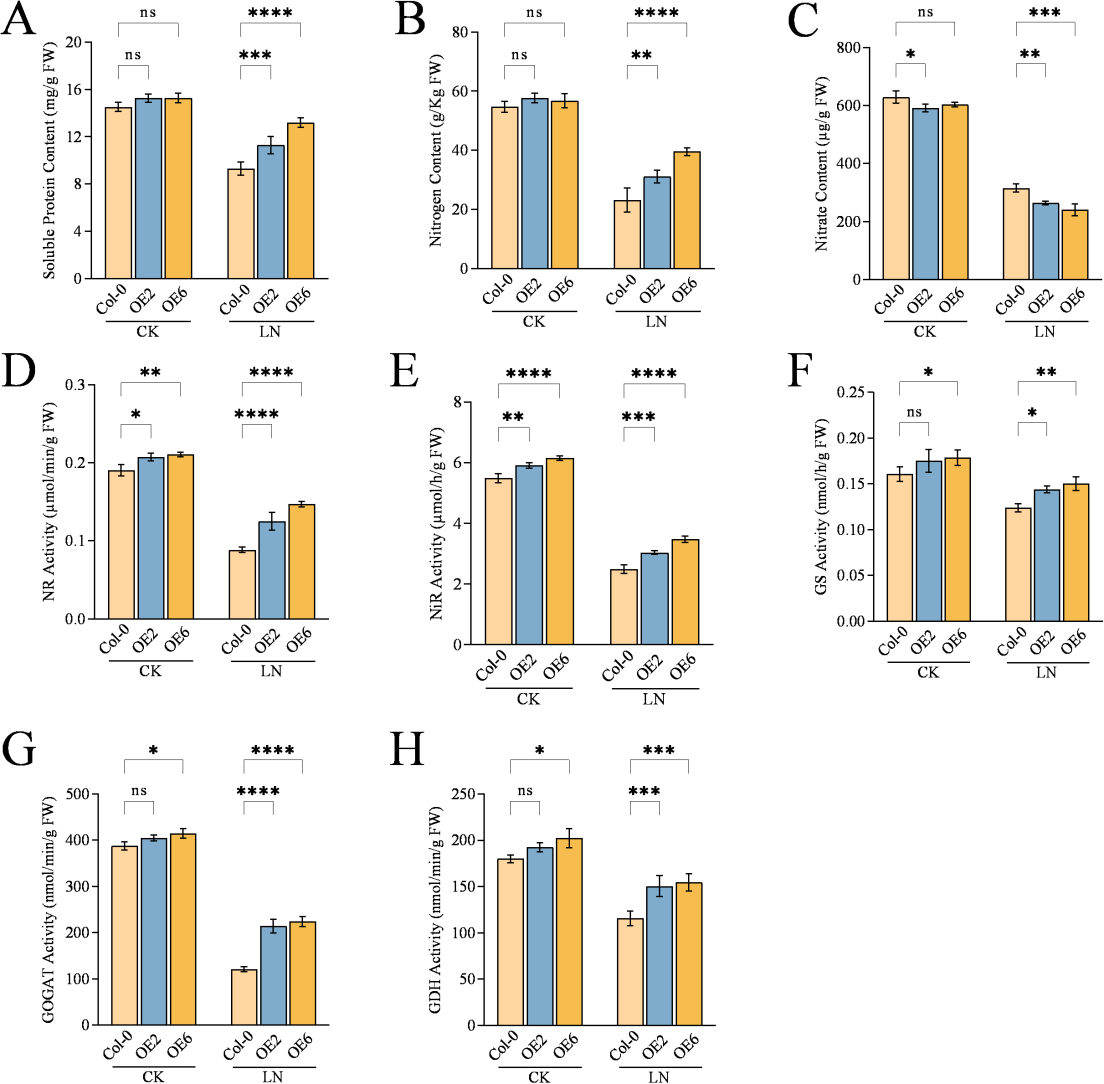
Overexpression of *CqNLP9* enhances nitrogen metabolism in *Arabidopsis* under LN. **(A)** Soluble protein content. **(B)** Nitrogen content. **(C)** Nitrate content. **(D)** Nitrate reductase (NR) activity, **(E)** Nitrite reductase (NiR) activity, **(F)** Glutamine synthase (GS) activity, **(G)** Glutamate synthase (GOGAT) activity, and **(H)** Glutamate dehydrogenase (GDH) activity. CK: complete nutrient solution; LN: low nitrogen solution. OE: *CqNLP9-*overexpressed lines (OE2 and OE6). Data are presented as means ± SD. Each biological replicate (n = 3 independent replicates) consisted of pooled leaf tissues from five individual plants per line per treatment. Statistical analysis was performed using two-way ANOVA followed by Duncan’s multiple range test. Asterisks indicate significant differences between the Col-0 and overexpression line (OE2 or OE6) within the same treatment group (*P < 0.05, **P < 0.01, ***P < 0.001, ****P < 0.0001; ns, not significant).

Soluble protein content reflects nitrogen metabolism status in plants. Under control (CK) conditions, no significant differences in soluble protein content were observed between Col-0 and the overexpression lines OE2 and OE6. After 30 days of low-nitrogen (LN) treatment, soluble protein content increased by 17.6% in OE2 (P = 0.0009) and 29.5% in OE6 (P < 0.0001) compared to Col-0 (**Figure 3A**).

Total nitrogen and nitrate content are key indicators of nitrogen assimilation. After 30 days of LN treatment, the total nitrogen content was 25.8% higher in OE2 (P = 0.0037) and 41.6% higher in OE6 (P < 0.0001) relative to Col-0 (**Figure 3B**). In contrast, under LN conditions, the nitrate content decreased by 16.19% in OE2 (P = 0.0035) and 23.8% in OE6 (P = 0.0002) compared to Col-0 (**Figure 3C**).

The role of *CqNLP9* on activities of nitrogen assimilation enzymes was assessed. After 30 days of LN treatment, all measured enzyme activities were significantly higher in OE2 and OE6 compared to Col-0. Specifically, nitrate reductase (NR) activity increased by 36% in OE2 (P < 0.0001) and 45.5% in OE6 (P < 0.0001) (**Figure 3D**), while nitrite reductase (NiR) activity increased by 17.6% in OE2 (P = 0.0001) and 28.2% in OE6 (P < 0.0001), respectively (**Figure 3E**). Glutamine synthetase (GS) activity in OE2 and OE6 was 1.19-fold (P = 0.0252) and 1.27-fold (P = 0.0045) higher than in Col-0 (**Figure 3F**). Glutamate synthase (GOGAT) activity was elevated 1.76-fold in OE2 (P < 0.0001) and 1.85-fold in OE6 (P < 0.0001) (**Figure 3G**), and glutamate dehydrogenase (GDH) activity increased by approximately 25.3% in both overexpression lines relative to Col-0 (**Figure 3H**).

**Figure 4:**
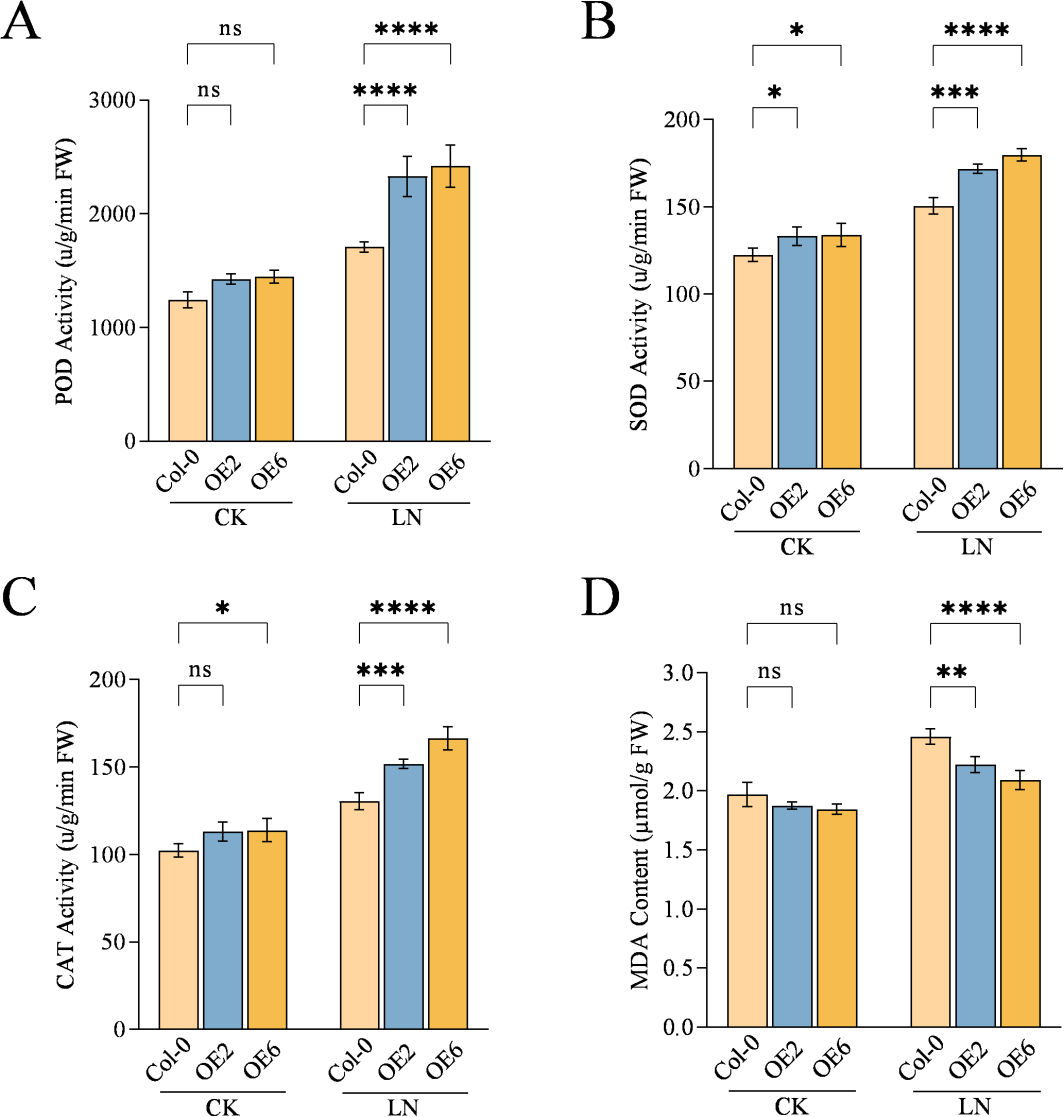
*CqNLP9* overexpression increased antioxidant activity under low-nitrogen conditions. **(A)** Peroxidase (POD) activity, **(B)** Superoxide dismutase (SOD) activity. **(C)** Catalase **(CAT)** activity. **(D)** Malondialdehyde (MDA) content. CK, Complete nutrient solution; LN, Low nitrogen solution. OE: *CqNLP9-*overexpressed lines (OE2 and OE6). Data are presented as means ± SD. Each biological replicate (n = 3 independent replicates) consisted of pooled leaf tissues from five individual plants per line per treatment. Statistical analysis was performed using two-way ANOVA followed by Duncan’s multiple range test. Asterisks indicate significant differences between the Col-0 and overexpression line (OE2 or OE6) within the same treatment group (*P < 0.05, **P < 0.01, ***P < 0.001, ****P < 0.0001; ns, not significant).

To investigate whether *CqNLP9* is involved in the reactive oxygen species (ROS) scavenging process under LN stress, we analyzed the activities of POD, SOD, and CAT, as well as the MDA content in three genotypes. After 30 days of the LN treatment, the activities of POD, SOD, and CAT in *CqNLP9*-OE lines were significantly elevated compared to Col-0. Specifically, POD activity increased by 36.3% in OE2 (P < 0.0001) and 41.5% in OE6 (P < 0.0001). SOD activity in *CqNLP9*-OE lines rose by approximately 20% (P = 0.0003) relative to Col-0. CAT activity increased by 16.2% in OE2 (P = 0.0009) and 27.7% in OE6 (P < 0.0001), respectively (**Figures 4A–C**). Additionally, after 30 days of treatment, MDA content in OE2 and OE6 was reduced by 17.8% (P = 0.0036) and 21.9% (P < 0.0001) compared to Col-0 (**Figure 4D**). These findings suggest that *CqNLP9* may enhance adaptation to low-nitrogen environments by modulating the antioxidant enzyme system and reducing cellular oxidative damage.

### 3.3 Silencing *NbNLP9* hinders nitrogen metabolism in tobacco

**Figure 5:**
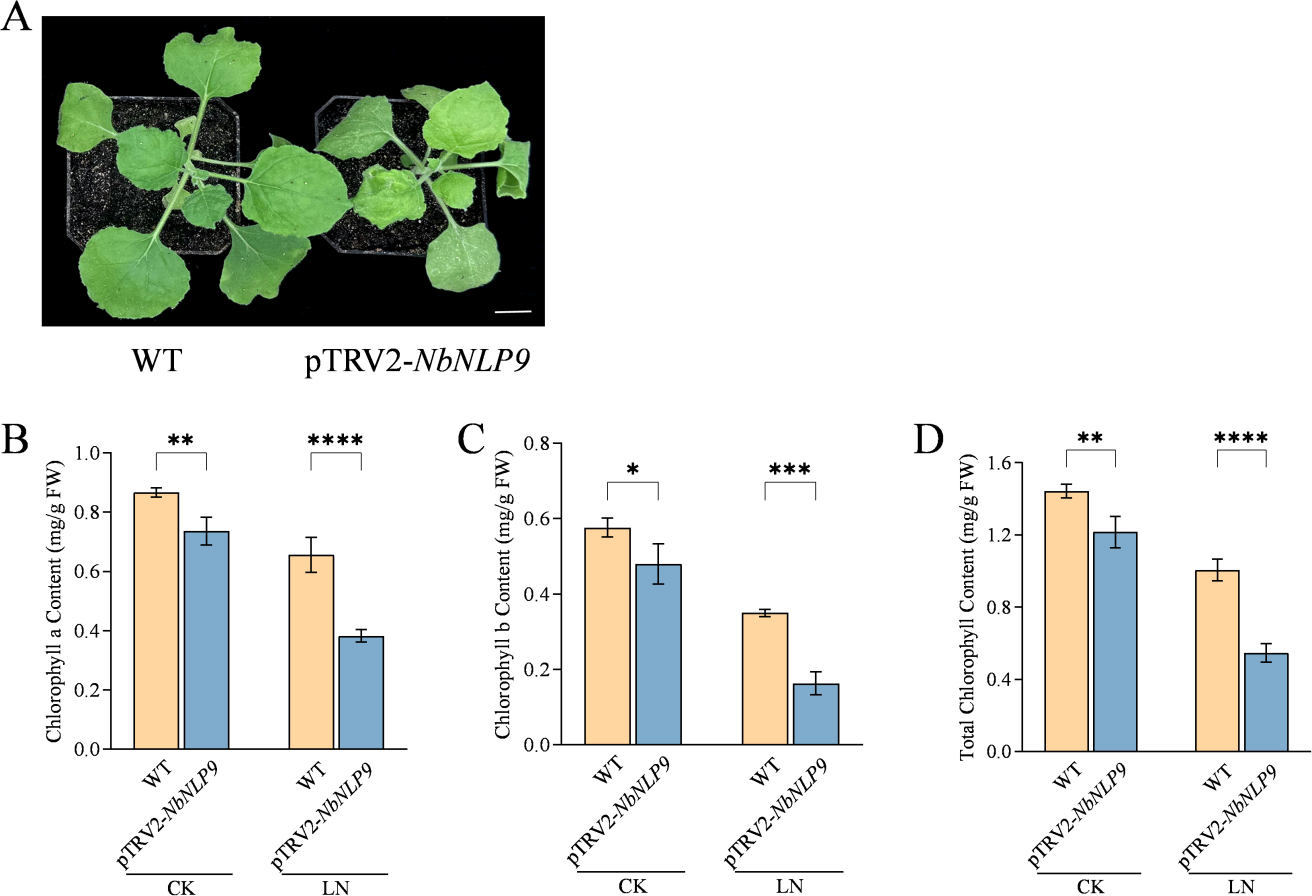
*NbNLP9* silencing impairs growth and chlorophyll biosynthesis in *Nicotiana benthamiana* under low-nitrogen conditions. **(A)** Growth of wild-type (WT) and *NbNLP9*-silenced (pTRV2-*NbNLP9*) plants after 30 days of low-nitrogen treatment. Scale bar = 1 cm. Three independent replicates were adopted, one replicate represented 10 single plant from each group. **(B)** Chlorophyll a content. **(C)** Chlorophyll b content. **(D)** Total chlorophyll content. CK, Complete nutrient solution; LN, Low nitrogen solution. WT, wild-type tobacco; pTRV2-*NbNLP9*: *NbNLP9*-silenced tobacco. Data are presented as means ± SD. Each biological replicate (n = 3 independent replicates) consisted of pooled leaf tissues from five individual plants per line per treatment. Statistical analysis was performed using two-way ANOVA followed by Duncan’s multiple range test. Asterisks indicate significant differences between the Col-0 and overexpression line (OE2 or OE6) within the same treatment group (*P < 0.05, **P < 0.01, ***P < 0.001, ****P < 0.0001; ns, not significant).

VIGS system was also adopted to verify *CqNLP9* biological function (**Supplementary Figures 2C, D**). After 30 days of the LN treatment, the silenced tobacco plants exhibited significantly stunted growth compared to the WT, with leaves displaying yellowing and wrinkling (**Figure 5A**). The fresh and dry weights of both the shoots and roots of the *NbNLP9*-silenced tobacco plants decreased markedly (**Supplementary Table 3**), which suggested that silencing the *NbNLP9* gene inhibits tobacco growth under LN stress. After 30 days of treatment, compared to WT tobacco, the chlorophyll a content in *NbNLP9*-silenced tobacco decreased by 47.9% (P < 0.0001), chlorophyll b content decreased by 66.7% (P = 0.0003), and total chlorophyll content decreased by 55.4% (P < 0.0001) (**Figures 5B–D**).

**Figure 6:**
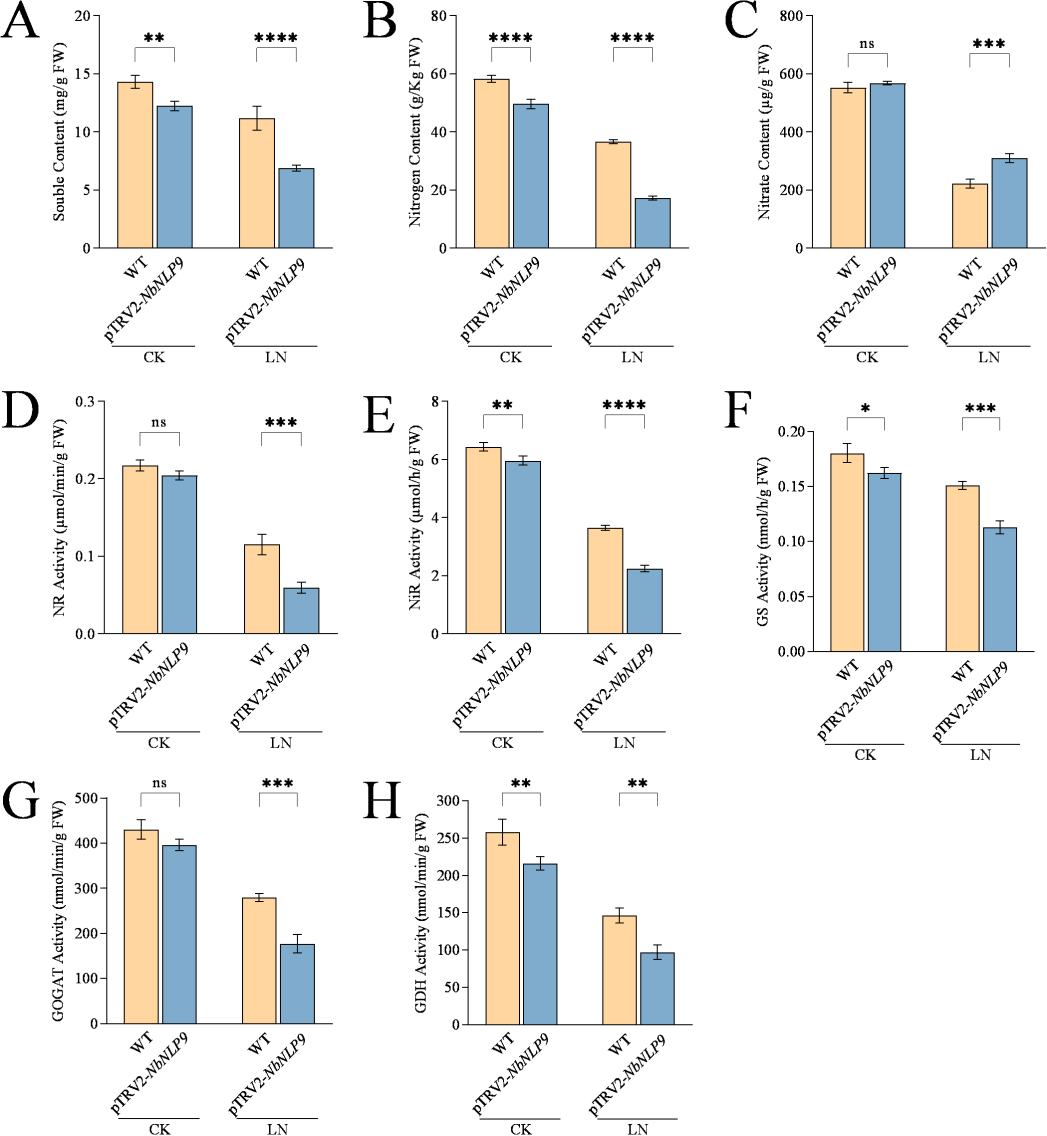
*NbNLP9* silencing disrupts nitrogen metabolism in *Nicotiana benthamiana*. **(A)** Soluble protein content. **(B)** Nitrogen content. **(C)** Nitrate content. **(D)** Nitrate reductase (NR) activity, **(E)** Nitrite reductase (NiR) activity, **(F)** Glutamine synthase (GS) activity, **(G)** Glutamate synthase (GOGAT) activity, and **(H)** Glutamate dehydrogenase (GDH) activity. CK, Complete nutrient solution; LN, Low nitrogen solution. WT, wild-type tobacco; pTRV2-*NbNLP9*: *NbNLP9*-silenced tobacco. Data are presented as means ± SD. Each biological replicate (n = 3 independent replicates) consisted of pooled leaf tissues from five individual plants per line per treatment. Statistical analysis was performed using two-way ANOVA followed by Duncan’s multiple range test. Asterisks indicate significant differences between the Col-0 and overexpression line (OE2 or OE6) within the same treatment group (*P < 0.05, **P < 0.01, ***P < 0.001, ****P < 0.0001; ns, not significant).

The soluble protein content declined in both WT and silenced plants under LN stress, with the soluble protein content in *NbNLP9*-silenced tobacco being lower than that in the WT (**Figure 6A**). The contents of total nitrogen and nitrate in WT and *NbNLP9*-silenced tobacco were compared. Under CK conditions, the total nitrogen content in the silenced plants decreased by 14.7% (P < 0.0001) and there was no significant difference in nitrate content. After 30 days of the LN treatment, the total nitrogen content in the silenced plants dropped by 53% (P < 0.0001), whereas the nitrate content increased by 28.3% (P = 0.0002) (**Figures 6B, C**). This suggests that the silencing of the *NbNLP9* gene might have diminished the plant’s capacity to assimilate nitrate.

Activities of key enzymes involved in nitrate metabolism were also analyzed. After 30 days of the LN treatment, NR activity in *NbNLP9*-silenced tobacco decreased by 54.5% (P = 0.0001) compared to WT, while NiR activity dropped by 38.1% (P < 0.0001) relative to WT tobacco (**Figures 6D, E**). After 30 days of treatment, the activities of GS, GOGAT and GDH were all significantly reduced in *NbNLP9*-silenced tobacco, following a similar trend to NR and NiR, with decreases of 25.2% (P = 0.0001), 36.7% (P = 0.0001), and 33.5% (P = 0.0019), respectively, compared to WT tobacco (**Figures 6F–H**).

**Figure 7:**
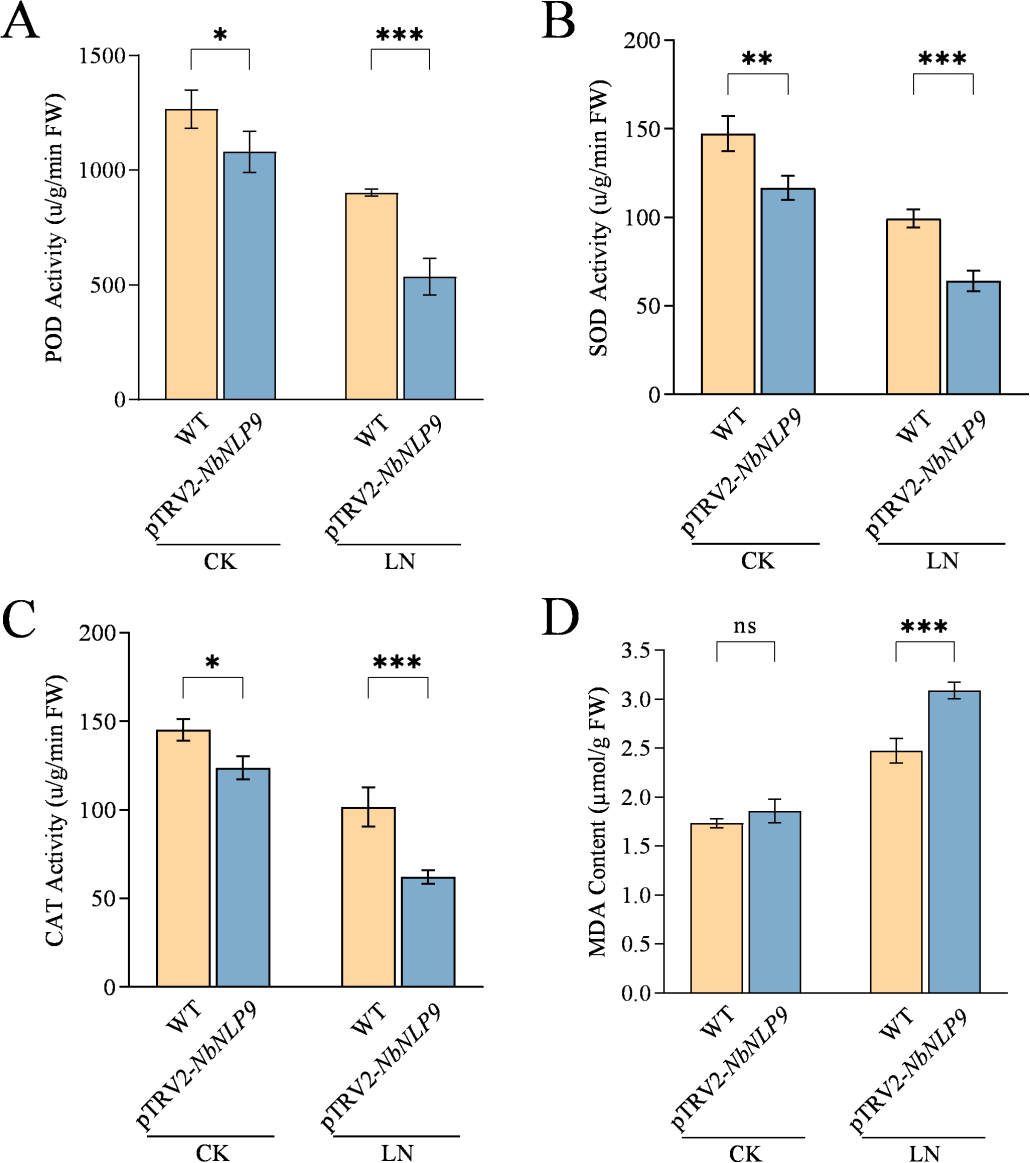
*NbNLP9* silencing depressed antioxidant enzyme and increased MDA content in *N. benthamiana* under low-nitrogen conditions. **(A)** Peroxidase (POD) activities, **(B)** Superoxide dismutase (SOD) activities. **(C)** Catalase (CAT) activities. **(D)** Malondialdehyde (MDA) content. CK, Complete nutrient solution; LN, Low nitrogen solution; WT, wild-type tobacco; pTRV2-*NbNLP9*: *NbNLP9*-silenced tobacco. Data are presented as means ± SD. Each biological replicate (n = 3 independent replicates) consisted of pooled leaf tissues from five individual plants per line per treatment. Statistical analysis was performed using two-way ANOVA followed by Duncan’s multiple range test. Asterisks indicate significant differences between the Col-0 and overexpression line (OE2 or OE6) within the same treatment group (*P < 0.05, **P < 0.01, ***P < 0.001; ns, not significant).

The impact of *NbNLP9* silencing on the antioxidant defense system were measured. After 30 days of treatment, activities of POD, SOD and CAT in *NbNLP9*-silenced tobacco decreased by 40.6% (P = 0.0006), 35.3% (P = 0.0006), and 38.6% (P = 0.0003), respectively, and MDA content increased by 19.8% (P = 0.0001) (**Figures 7A–D**).

### 3.4 CqNLP9 localization and interactions with CqMOB1B

**Figure 8:**
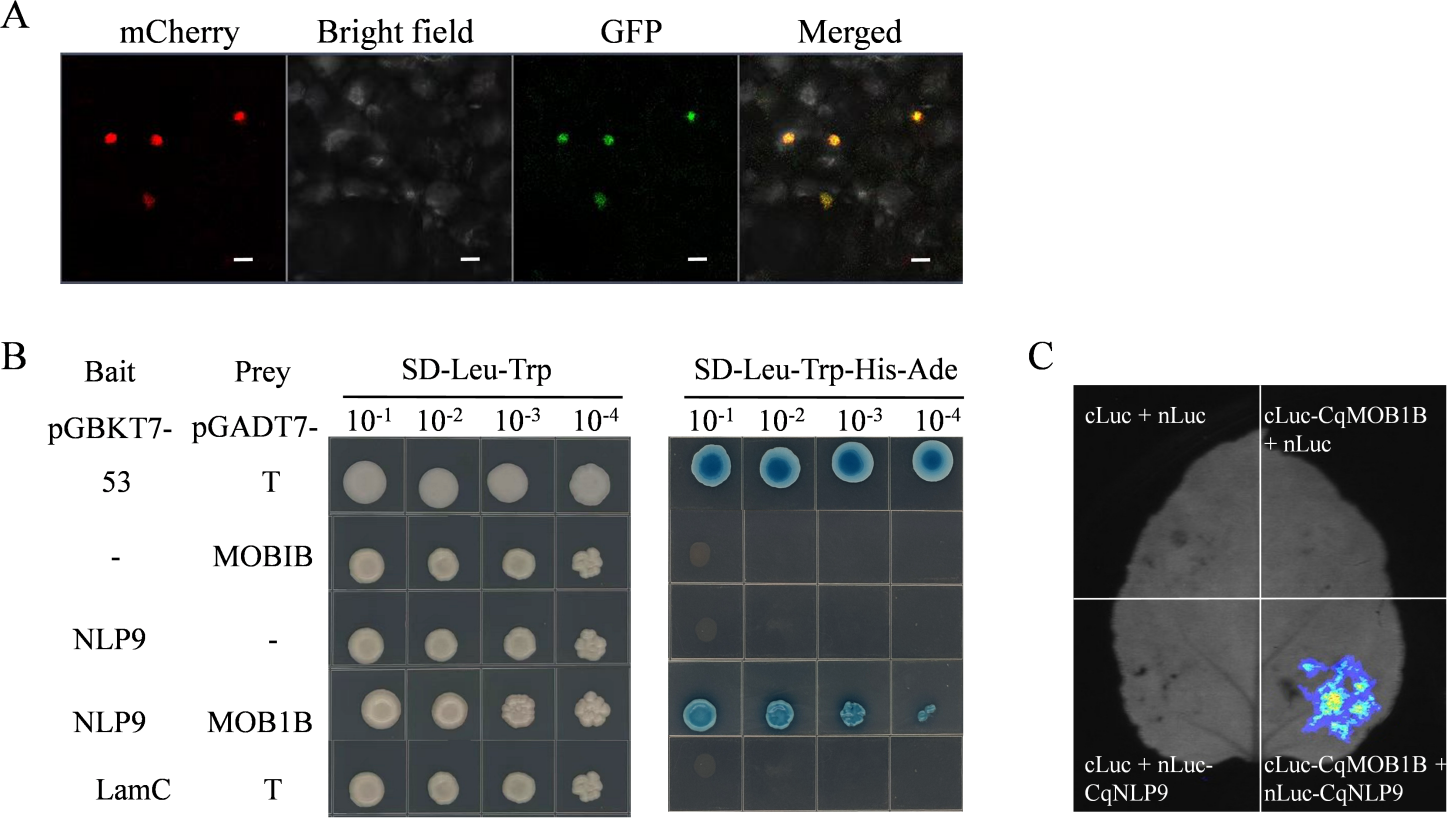
Protein-protein interaction between CqNLP9 and CqMOB1B. **(A)** Subcellular localization of CqNLP9 in *N. benthamiana* epidermal cells. Confocal images show co-localization of CqNLP9-GFP (green) with nuclear marker H2B-mCherry (red). Scale bar = 50 µm. **(B)** Yeast two-hybrid assay. Co-transformation of pGBKT7-*CqNLP9* (bait) and pGADT7-*CqMOB1B* (prey) in yeast on SD/-Leu/-Trp/-His/-Ade medium. Positive control: pGADT7-*T* + pGBKT7-*53*; negative controls: empty vectors. **(C)** Dual-molecule luciferase complementation (LUC) assay. Control groups: cLuc + nLuc, cLuc + nLuc-CqNLP9, cLuc-CqMOB1B + nLuc; Experimental group: cLuc-CqMOB1B + nLuc-CqNLP9.

To determine the subcellular localization of the CqNLP9, we successfully constructed a pSUPER1300-GFP-*CqNLP9* recombinant plasmid (**Supplementary Figure 1C**). Co-expression of pSUPER1300-GFP-*CqNLP9* and the nuclear marker *H2B*-mCherry in *N. benthamiana* leaves demonstrated nuclear co-localization via confocal microscopy (**Figure 8A**).

To verify potential protein-protein interactions between CqNLP9 and CqMOB1B, we conducted yeast two-hybrid (Y2H) and luciferase complementation (LUC) experiments *in vitro* and *in vivo*. On SD/-Leu/-Trp selective solid medium, all yeast strains grew normally. On SD/-Leu/-Trp/-His/-Ade quadruple-selective solid medium, the combinations of pGADT7-*T* + pGBKT7–*53* and pGADT7-*CqMOB1B* + pGBKT7-*CqNLP9* exhibited normal growth, and the yeast colonies turned blue after the addition of X-α-gal dye. In contrast, the growth of yeast in the combinations of pGADT7 + pGBKT7-*CqNLP9* and pGBKT7 + pGADT7-*CqMOB1B* was inhibited (**Figure 8B**). These results preliminarily confirmed an interaction between CqNLP9 and CqMOB1B.

We further performed a luciferase complementation (LUC) imaging assay in *Nicotiana benthamiana* leaves to validate the protein-protein interaction *in vivo*. In this system, reconstitution of the functional N- and C-terminal luciferase fragments results in luminescence upon substrate addition. As shown in **Figure 8C**, a clear luminescent signal was detected only when CqMOB1B and CqNLP9 were co-expressed, while control combinations lacking either partner showed no detectable signal. These results confirm the specific interaction between CqNLP9 and CqMOB1B in plant cells.

## 4 Discussion

Nitrogen availability critically regulates plant growth and development. *NLP* transcription factors function as central regulators of nitrate signaling and assimilation (Riveras et al., 2015; Liu et al., 2022; Schenk et al., 2025). Abundant studies have shown *NLP* transcription factors regulate the nitrogen assimilation under nitrogen deficient conditions (Zhang et al., 2022; Durand et al., 2023; Kim et al., 2023). In this study, the role of *CqNLP9* in low nitrogen tolerance was investigated using heterologous systems—*Arabidopsis* overexpression and tobacco VIGS—due to the current lack of stable genetic transformation methods in quinoa. In *Arabidopsis*, *CqNLP9* overexpression enhanced LN tolerance, while silencing its tobacco ortholog *NbNLP9* increased LN sensitivity. These conserved phenotypic responses across two distantly related species suggest that *CqNLP9* may contribute to nitrogen stress adaptation in its native context. This interpretation is further supported by the strong induction of *CqNLP9* expression in quinoa under LN stress (**Figure 1A**).

*CqNLP9* improves nitrogen metabolism under LN. In plants, nitrate reductase (NR) and nitrite reductase (NiR) catalyze the sequential reduction of NO_3_^-^ to NH_4_^+^, a process strongly influenced by nitrogen availability (Guo et al., 2014; Balotf et al., 2016). The GS/GOGAT cycle represents the primary route for NH_4_^+^ assimilation in plants, with GOGAT playing a dominant role in nitrogen accumulation and remobilization (Liu et al., 2016). Glutamate dehydrogenase (GDH) functions supplementarily, mainly in ammonium detoxification due to its low affinity for NH_3_ (Lea et al., 1992; Melo-Oliveira et al., 1996). In this study, the activities of nitrogen assimilation enzymes were decreased in wild-type *Arabidopsis* and *NbNLP9*-silenced tobacco plants. While *CqNLP9*-overexpressing lines exhibited elevated activities of nitrogen assimilation enzymes. This aligns with the established role of *NLP* transcription factors as nitrate sensors that promote efficient nitrogen utilization under nitrogen-limited conditions (Yanagisawa, 2014; Jagadhesan et al., 2020). It was reported LN stress may alter plant physiology, affecting nitrate transporter function on root cell membranes and limiting nitrate uptake (Zhu et al., 2024). An intriguing observation in this study is the inverse relationship between nitrate content and total nitrogen in *CqNLP9*-overexpressing lines under LN stress. If the reduced nitrate level were primarily due to decreased uptake or a growth dilution effect, total nitrogen would not be expected to increase. The concurrent elevation of total nitrogen and assimilation enzyme activities therefore supports enhanced nitrate assimilation capacity in *CqNLP9*-overexpressing lines. While we acknowledge that tissue nitrate levels are influenced by multiple factors, including uptake, vacuolar storage, and transport. Therefore, although our data strongly suggest improved nitrogen assimilation capacity, definitive proof of enhanced assimilation efficiency will require direct flux measurements using ^15^N-labeled nitrate in future investigations.

*CqNLP9* alleviated oxidative stress induced by low-nitrogen (LN) conditions. Under LN stress, plants typically accumulate reactive oxygen species (ROS), which can damage proteins, disrupt membrane integrity, and elevate malondialdehyde (MDA) levels—a marker of lipid peroxidation (Møller et al., 2007; Sahu et al., 2022). Antioxidant enzymes such as superoxide dismutase (SOD), peroxidase (POD), and catalase (CAT) constitute a key enzymatic ROS-scavenging system that helps maintain redox homeostasis (Kumar et al., 2023). Herein, *CqNLP9*-overexpressing lines exhibited significantly higher activities of SOD, POD, and CAT under LN stress compared to wild-type (Col-0) plants, along with reduced MDA accumulation (**Figure 4**). In contrast, *NbNLP9*-silenced tobacco plants showed the opposite trend: decreased antioxidant enzyme activities and elevated MDA content (**Figure 7**). These results indicate that *CqNLP9* enhances the capacity of the antioxidant enzyme system under LN, thereby limiting ROS-mediated membrane damage and supporting cellular redox balance. This finding aligns with earlier reports linking *NLP*-family transcription factors to improved oxidative stress tolerance under nutrient limitation (Safi et al., 2021; Wang et al., 2025a). Together, the enhanced antioxidant defense system contributes to low-nitrogen (LN) tolerance in *CqNLP9*-overexpressing plants, supporting its function in improving nitrogen assimilation under stress.

In plants, MOB1 functions as a core component of the Hippo signaling pathway and has been implicated in the regulation of programmed cell death (Citterio et al., 2005; Citterio et al., 2006). In this study, we investigated whether CqNLP9 interacts with CqMOB1B. Yeast two-hybrid and luciferase complementation assays confirmed a direct protein-protein interaction between CqNLP9 and CqMOB1B. This finding suggests a potential interaction between a nitrogen-responsive transcription factor and a Hippo pathway core component in plants. However, this interaction requires further experimental validation. Future studies employing orthogonal approaches such as co-immunoprecipitation (co-IP) and bimolecular fluorescence complementation (BiFC) will help confirm the interaction between CqNLP9 and CqMOB1B.

Herein, the role of *CqNLP9* in low-nitrogen tolerance was investigated using heterologous systems—*Arabidopsis* overexpression and tobacco VIGS—due to the current lack of stable genetic transformation methods in quinoa. However, direct functional evidence in quinoa itself remains necessary to definitively establish its role. Future studies using transient assays or, once available, stable genome editing in quinoa will help confirm whether the mechanisms observed in heterologous systems operate similarly in the native host.

## 5 Conclusions

In conclusion, our study proposed that the quinoa transcription factor *CqNLP9* functions as a key regulator of plant adaptation to low-nitrogen (LN) stress. *CqNLP9* seems to improve LN tolerance through a dual mechanism: (1) by upregulating key enzymes involved in nitrogen uptake and assimilation, thereby improving nitrogen-use efficiency; and (2) by reinforcing the antioxidant system, which mitigates oxidative damage under LN conditions. Furthermore, the physical interaction between CqNLP9 and CqMOB1B—a core component of the Hippo signaling pathway—uncovers a previously unrecognized molecular interface that potentially links nitrogen sensing to developmental regulation in plants. These findings not only deepen our mechanistic understanding of nitrogen-stress adaptation in quinoa but also highlight *CqNLP9* as a promising candidate gene for breeding crops with improved nitrogen-use efficiency and sustained performance under nitrogen-limited environments.

## Data Availability

The raw data supporting the conclusions of this article will be made available by the authors, without undue reservation.
