# Supplementary material for: Quinoa CqNLP9: a possible regulator of nitrogen metabolism under low nitrogen stress
Source: Front Plant Sci. 2026 Mar 18;17:1787306. doi: 10.3389/fpls.2026.1787306 (PMC13038593; doi:10.3389/fpls.2026.1787306)
Supplement: Supplementary file 1 [file SupplementaryFile1.docx]

Supplementary Material

**Supplemental Table 1.** Components of nutrient solution.

| Components | Concentrations | |
| --- | --- | --- |
|  | complete nutrient solution (CK) | low-nitrogen solution (LN) |
| Ca(NO_3_)_2_ | 0.5 mol/L | 0.1 mol/L |
| KNO_3_ | 0.5 mol/L | 0.1 mol/L |
| KH_2_PO_4_ | 2.3 mmol/L | 2.3 mmol/L |
| MgSO_4_·7H_2_O | 2.5 mmol/L | 2.5 mmol/L |
| Fe-EDTA | 0.02 mmol/L | 0.02 mmol/L |
| H_3_BO_3_ | 0.05 mmol/L | 0.05 mmol/L |
| MnSO_4_ | 7 μmol/L | 7 μmol/L |
| CuSO_4_·5H_2_O | 0.3 μmol/L | 0.3 μmol/L |
| ZnSO_4_ | 0.8 μmol/L | 0.8 μmol/L |
| H_2_MoO_4_ | 0.5 μmol/L | 0.5 μmol/L |

**Supplemental Table 2.** Growth profile of *Arabidopsis* strains Col-0, *CqNLP9*-OEs after 30 days under low-nitrogen (LN) stress.

|  | Col-0 | *CqNLP9-*OE2 | *CqNLP9-*OE6 |
| --- | --- | --- | --- |
| Plant Heigh (cm) | 14.6 ± 2.3 | 20.8 ± 1.1^*^ | 21.2 ± 0.8^*^ |
| Root Length (cm) | 9.2 ± 2.1 | 12.6 ± 2.3^*^ | 12.9 ± 1.5^*^ |
| Shoot Fresh Weight (mg) | 225.8 ± 4.527 | 403.9 ± 9.647^*^ | 427 ± 12.686^*^ |
| Root Fresh Weight (mg) | 17.8 ± 0.8 | 22.5 ± 0.9^*^ | 23.6 ± 0.7^*^ |
| Shoot Dry Weight (mg) | 16.35 ± 1.4 | 22.56 ± 1.9^*^ | 23.35 ± 2.3^*^ |
| Root Dry Weight (mg) | 1.88 ± 0.09 | 2.7 ± 0.11^*^ | 2.91 ± 0.13^*^ |

**Note:** Individual plants from each genotype were measured and weighed separately. Data are means ± SD (n = 10). Differences among the same index were analyzed by two-way ANOVA, Duncan’s multiple range test, *significant difference at P < 0.05.

**Supplemental Table 3.** Biomass of wild-type (WT) and *NbNLP9*-silenced tobacco under low-nitrogen (LN) conditions.

|  | WT | pTRV2-*NbNLP9* |
| --- | --- | --- |
| Shoot Fresh Weight (g) | 2.48 ± 0.13 | 1.36 ± 0.09^*^ |
| Root Fresh Weight (g) | 0.49 ± 0.011 | 0.33 ± 0.010^*^ |
| Shoot Dry Weight (mg) | 238 ± 8.1 | 133.2 ± 6.4^*^ |
| Root Dry Weight (mg) | 44.1 ± 0.9 | 31.7 ± 0.6^*^ |

**Note:** Individual plants from each strain were measured and weighed separately. Data are means ± SD (n = 10). Difference between the same index was analyzed by two-way ANOVA, Duncan’s multiple range test, *significant difference at P < 0.05.

**Supplemental Figure 1. Schematic diagram of vector construction.** (A) Schematic diagram of the pBI121-*CqNLP9* overexpression vector, where the *CqNLP9* gene is cloned into the pBI121 vector via double digestion with *Bam*H Ⅰ/*Sam* Ⅰ. (B) Schematic diagram of the pTRV2-*NbNLP9* silencing vector, where the *NbNLP9* gene is cloned into the pTRV2 vector via double digestion with *Xba* Ⅰ/*Bam*H Ⅰ. (C) Schematic diagram of the pSUPER1300-GFP-*CqNLP9* vector using *Sal* Ⅰ/*Kpn* Ⅰ double digestion to observe the subcellular localization of CqNLP9.

**Supplemental Figure 2. Obtaining of homozygous T3 *CqNLP9*-OE lines and tobacco with silenced *NbNLP9.*** (A) *Arabidopsis* transgenic lines heterologously expressing *CqNLP9*. (B) Using *AtACT* as the internal reference gene, verification of *CqNLP9* transcription in Col-0, OE2, and OE6 by RT-PCR. Three independent replicates were used. (C) The phenotypes of WT, the control group pTRV2-*PDS*, and the experimental group pTRV2-*NbNLP9* tobacco. (D) Using *NbActin* as the internal reference gene, verification of *NbNLP9* expression in WT, pTRV2-*PDS*, and pTRV2-*NbNLP9* tobacco by RT-PCR. Experiments were repeated with three independent biological samples.
